# Genome-wide identification of transcriptional start sites in the haloarchaeon *Haloferax volcanii* based on differential RNA-Seq (dRNA-Seq)

**DOI:** 10.1186/s12864-016-2920-y

**Published:** 2016-08-12

**Authors:** Julia Babski, Karina A. Haas, Daniela Näther-Schindler, Friedhelm Pfeiffer, Konrad U. Förstner, Matthias Hammelmann, Rolf Hilker, Anke Becker, Cynthia M. Sharma, Anita Marchfelder, Jörg Soppa

**Affiliations:** 1 Institute for Molecular Biosciences, Goethe University, Biocentre, Max-von-Laue-Str. 9, D-60439 Frankfurt, Germany; 2 Biologie II, University of Ulm, 89069 Ulm, Germany; 3 Computational Biology Group, MaxPlanckInstitute of Biochemistry, Am Klopferspitz 18, 82152 Martinsried, Germany; 4 Research Center for Infectious Diseases (ZINF), University of Würzburg, Josef-Schneider-Str. 2/D15, 97080 Würzburg, Germany; 5 Bioinformatik und Systembiologie, University of Gießen, Heinrich-Buff-Ring 58, 35392 Gießen, Germany; 6 LOEWE-Center for Synthetic Microbiology, Hans-Meerwein-Str., 35032 Marburg, Germany

**Keywords:** Archaea, *Haloferax volcanii*, dRNA-Seq, Transcriptome, Promoter, Leaderless transcript, Non-coding RNA, sRNA, Antisense RNA

## Abstract

**Background:**

Differential RNA-Seq (dRNA-Seq) is a recently developed method of performing primary transcriptome analyses that allows for the genome-wide mapping of transcriptional start sites (TSSs) and the identification of novel transcripts. Although the transcriptomes of diverse bacterial species have been characterized by dRNA-Seq, the transcriptome analysis of archaeal species is still rather limited. Therefore, we used dRNA-Seq to characterize the primary transcriptome of the model archaeon *Haloferax volcanii*.

**Results:**

Three independent cultures of *Hfx. volcanii* grown under optimal conditions to the mid-exponential growth phase were used to determine the primary transcriptome and map the 5′-ends of the transcripts. In total, 4749 potential TSSs were detected. A position weight matrix (PWM) was derived for the promoter predictions, and the results showed that 64 % of the TSSs were preceded by stringent or relaxed basal promoters. Of the identified TSSs, 1851 belonged to protein-coding genes. Thus, fewer than half (46 %) of the 4040 protein-coding genes were expressed under optimal growth conditions. Seventy-two percent of all protein-coding transcripts were leaderless, which emphasized that this pathway is the major pathway for translation initiation in haloarchaea. A total of 2898 of the TSSs belonged to potential non-coding RNAs, which accounted for an unexpectedly high fraction (61 %) of all transcripts. Most of the non-coding TSSs had not been previously described (2792) and represented novel sequences (59 % of all TSSs). A large fraction of the potential novel non-coding transcripts were cis-antisense RNAs (1244 aTSSs). A strong negative correlation between the levels of antisense transcripts and cognate sense mRNAs was found, which suggested that the negative regulation of gene expression via antisense RNAs may play an important role in haloarchaea. The other types of novel non-coding transcripts corresponded to internal transcripts overlapping with mRNAs (1153 iTSSs) and intergenic small RNA (sRNA) candidates (395 TSSs).

**Conclusion:**

This study provides a comprehensive map of the primary transcriptome of *Hfx. volcanii* grown under optimal conditions. Fewer than half of all protein-coding genes have been transcribed under these conditions. Unexpectedly, more than half of the detected TSSs belonged to several classes of non-coding RNAs. Thus, RNA-based regulation appears to play a more important role in haloarchaea than previously anticipated.

**Electronic supplementary material:**

The online version of this article (doi:10.1186/s12864-016-2920-y) contains supplementary material, which is available to authorized users.

## Background

A transcriptome, by definition, encompasses the complete set of RNA within an organism. Transcriptomes are highly dynamic, especially in prokaryotes, and change in response to varying environmental conditions, growth stages, or developmental states. Transcriptomes were initially thought to be nearly entirely composed of rRNAs, tRNAs, several other housekeeping RNAs, and protein-coding mRNAs. In recent years, research has shown that eukaryotes as well as prokaryotes contain a large number of additional non-coding RNAs. In prokaryotes, these non-coding regulatory RNAs are called “sRNAs” (or “ncRNAs”), and they range in size from approximately 50 to 500 nt [1–4]). The application of high-throughput sequencing for the analysis of cDNA libraries (RNA-Seq) and specialized variants has enabled a comprehensive overview of bacterial and archaeal transcriptomes and led to the discovery of many new sRNAs in all studied species [5–8].

Although a number of RNA-Seq studies of bacterial species have been published in recent years, the number of studies on the transcriptomes of archaea is still limited. Until now, RNA-Seq analyses have only been performed with nine archaeal species: *Methanosarcina mazei*, *Sulfolobus solfataricus*, *Halobacterium salinarum*, *Haloferax volcanii*, *Nanoarchaeum equitans*, *Methanopyrus kandleri*, *Pyrococcus abyssi*, *Thermococcus kodakarensis*, and *Methanolobus psychrophilus* [9–16].

*Hfx. volcanii* is a model archaeal species used to study many central biological processes, including replication, DNA repair, transcription and transcriptional regulation, translation, protein export, posttranslational protein modification, protein degradation, metabolism, and the CRISPR-Cas system [17–24]. *Hfx. volcanii* can easily be cultivated in synthetic or complex media under aerobic or anaerobic conditions and has a generation time of approximately 3 h under optimal conditions [25]. The genome of *Hfx. volcanii* has been sequenced and consists of one major chromosome (2.9 Mbp), three smaller chromosomes (from 89 to 690 kbp), and one smaller plasmid (6.4 kbp) [26]. Functional genomic techniques for analyzing transcriptomes, proteomes, and metabolomes have been established [27]. A transformation protocol was described in 1987 [28], and since then, many genetic tools and techniques have been developed, such as for the rapid and easy generation of in-frame deletion mutants [29–31].

Several characteristic features of the *Hfx. volcanii* transcriptome have been analyzed in small-scale studies. One study identified the 5′-ends of 62 haloarchaeal transcripts and determined that the majority did not have 5′-UTRs and were thus leaderless [32]. The first regulatory sRNAs were identified in 2009 using an RNomics approach [33]. Additional sRNAs were identified using bioinformatics predictions [34]. The construction and phenotyping of a set of 27 sRNA gene deletion mutants demonstrated the importance of *Hfx. volcanii* sRNAs for many biological functions [35]. An RNA-Seq based analysis of the sRNA fraction (17 to 500 nt) increased the number of known *Hfx. volcanii* sRNAs to nearly 200 [11]. However, because of the limitations of RNA-Seq methodology at that time, the analysis was not comprehensive and only focused on small RNAs without providing a complete overview of the transcriptome. The present study obtained greater transcriptome coverage by combining state-of-the-art technology and the dRNA-Seq approach for analyzing the Hfx. volcanii primary transcriptome.

The dRNA-Seq approach allows for a global identification of transcriptional start sites (TSSs) at a single-nucleotide resolution [8, 36]. This approach is based on the differential sequencing of two cDNA libraries: one generated from untreated RNA and another treated with 5′P-dependent terminator exonuclease (Terminator EXonuclease, TEX). TEX digests RNAs with 5′-monophosphates, whereas primary transcripts with 5′-triphosphates are not degraded. Therefore, the sequencing of TEX−/+ libraries leads to a greater enrichment of primary transcripts in the TEX+ library compared with that of the untreated sample (TEX-), and the corresponding changes in the read patterns can be used to annotate the primary 5′-ends of the transcripts. The dRNA-Seq approach has been applied to various bacterial species [8] but has only been applied to two archaeal species thus far [9, 15].

## Results and discussion

### dRNA-Seq and classification of TSSs

To globally identify TSSs in *Hfx. volcanii*, dRNA-Seq was performed using *Hfx. volcanii* cultures grown to the mid-exponential growth phase under optimal conditions. Three independent cultures were grown, and the results were pooled for data analysis. In total, 200 million reads were obtained by Illumina sequencing and mapped to the genome sequence of *Hfx. volcanii*. Statistics related to sequencing and mapping are summarized in Additional file 1: Table S1.

In total, 4749 TSSs were identified in at least one dataset, 1493 TSSs were identified in at least two datasets, and 1191 TSSs were identified in all three datasets. The identified TSSs were equally distributed on both strands of the genome. Additional file 2: Table S2 lists all of the identified TSSs for *Hfx. volcanii* and includes additional information (discussed below), including their genomic localization, associated genes, affiliation with different transcript classes, read coverage, and promoter scores. For each transcript, the table also lists in which of the three experiments the transcript was detected and in which it was predicted to be a primary transcript based on the enrichment after TEX treatment.

The TSSs were grouped into the following classes according to their gene associations: 1) protein-coding transcripts, 2) previously known non-coding RNAs, and 3) novel transcripts (Table 1). The protein-coding transcripts were subdivided into leaderless transcripts and transcripts with 5′-UTRs. The novel transcripts were subdivided into intergenic RNAs and RNAs overlapping known genes in either the antisense (aTSS) or sense orientation. The latter were termed internal TSSs (iTSSs), which is consistent with earlier publications [15]. A generally accepted universal nomenclature for TSSs is not currently available [37].

**Table 1:** Classification of primary transcriptional start sites

Transcriptional start sites (TSSs)	Chr	pHV1	pHV3	pHV4	∑	Percent
All	3248	149	510	842	**4749**	
Forward	1646	69	263	414	2392	50
Reverse	1602	80	247	428	2357	50
Protein-coding gene TSSs	1421	32	170	228	**1851**	
Leaderless	1068	19	106	136	1329	72
With leader	353	13	64	92	522	28
Known RNAs^a^	92	2	6	6	**106**	
Novel gene TSSs	1735	115	334	608	**2792**	
Intergenic	239	14	57	85	395	14
Antisense (aTSS)	764	61	126	293	1244	45
Internal (iTSS)	732	40	151	230	1153	41

^a^validated sRNAs prepublished in Heyer et al., 2012, as well as tRNAs, rRNAs, 7S RNA, RNAse P RNA, and crRNAs (annotations taken from HaloLex)

The numbers of all transcription start sites and that of the three major classes are shown in bold

### Primary transcripts of protein-coding genes

In total, 1851 transcripts for protein-coding genes were detected, which corresponded to 46 % of the 4040 protein-coding genes annotated in the *Hfx. volcanii* genome. The *Hfx. volcanii* cultures were grown under optimal conditions; therefore, many functions were not required, such as enzymes involved in many biosynthetic pathways and proteins involved in stress adaptation, biofilm formation and chemotaxis. In addition, because a number of proteins are typically encoded in operons (e.g., ribosomal proteins, ABC transporters, chemotaxis proteins, and heteromeric enzymes), certain TSSs belonged to polycistronic transcripts that encode more than one protein (e.g., five proteins for ABC transporters operons). In a separate study, an in-depth analysis of the *Hfx. volcanii* proteome under standard growth conditions during the exponential growth phase identified 2360 proteins (Jevtic Z, Stoll B, Sharma K, Urlaub H, Marchfelder A, Lenz, C. and Pfeiffer F, in preparation). Because certain TSSs belonged to polycistronic transcripts, the 1851 TSSs that were identified by dRNA-Seq were consistent with the 2360 proteins that were identified using proteomics.

Wide variations have been observed in the fractions of mRNAs detected in other archaea using (d)RNA-Seq, with 20 % of the transcripts from protein-coding genes detected in *M. mazei* [9], 89.5 % detected in *Sulfolobus solfataricus* [10], and 92 % detected in *Methanolobus psychrophilus* [16]. However, the number of predicted TSSs was highly influenced by the growth conditions, the sequencing depth, and the program and parameters used for automatic TSS prediction, among other factors.

**Fig. 1:**
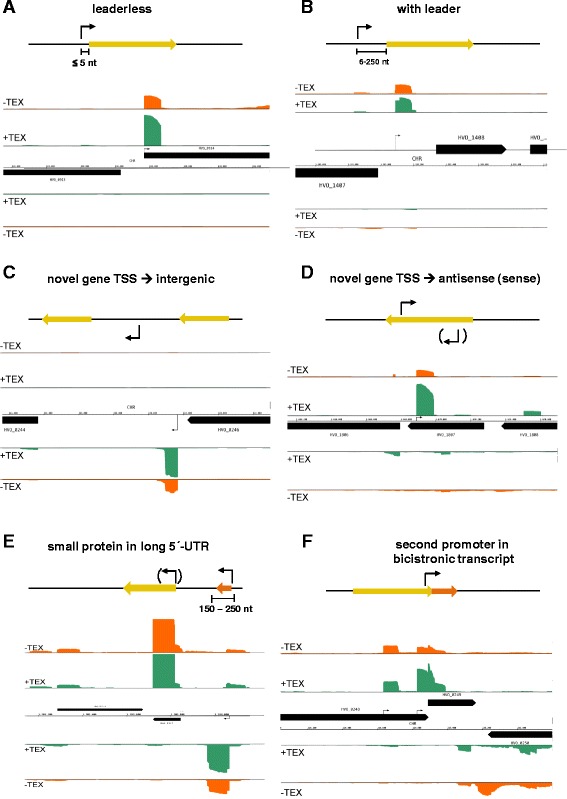
Classification of transcription start sites (TSS) in *Hfx. volcanii.* **a**-**f** In each case, a schematic overview is shown above a screenshot of the dRNA-Seq data in the Genome Browser. The name of the TSS class is shown on *top. Yellow bars* indicate protein-coding genes. *Black arrows* represent the transcription start sites (TSS). Screenshots: *Green* (+TEX) and *red* (−TEX) regions represent dRNA-Seq reads, and the height corresponds to the coverage. Reads mapping to the top strand are shown above the line representing the genome, reads mapping to the bottom strand are shown below the line. The same is true for the *black bars* representing annotated genes

The transcripts of protein-coding genes were subdivided into leaderless transcripts and transcripts with 5′-UTRs. Based on previous small-scale studies [32, 38], leaderless transcripts were defined as those containing zero to five nucleotides upstream of the start codon; therefore, the class of transcripts with leaders containing 5′-UTRs ranged in size from 6 to 250 nt. Schematic overviews and representative dRNA-Seq results for both classes are shown in Fig. 1a and b.

#### Leaderless transcripts

**Fig. 2:**
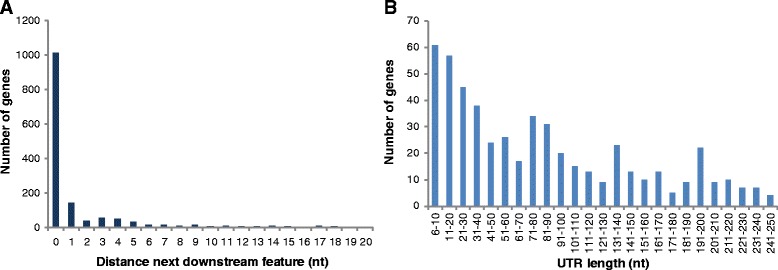
Leaderless and leadered transcripts in *Hfx. volcanii*. **a** Number of genes with zero to 20 additional nucleotides between the experimentally determined TSS and the annotated start codon. **b** Number of genes with 6 to 250 nucleotides between the experimentally determined TSS and the annotated start codon are shown in distance groups of ten nucleotides

To analyze whether the applied cutoff for leaderless transcripts was validated by the large dataset of this dRNA-Seq study, the number of protein-coding transcripts with zero to 20 additional nucleotides was tabulated (Fig. 2a). Sensu stricto leaderless transcripts that started on the translational start codon represented most of the leaderless transcripts with more than 1000 cases (approximately 80 % of all leaderless transcripts). Transcripts with one to five additional nucleotides were identified in 317 cases, whereas transcripts with six or more additional nucleotides were rare. Therefore, these results reinforced the earlier definition of leaderless transcripts described above, and the definition remained unchanged. Taken together, 72 % of all protein-coding transcripts were leaderless, which highlighted that translation initiation on leaderless transcripts was the major pathway in halophilic archaea.

Not all archaea contain mostly leaderless transcripts. In fact, archaeal transcriptomes are highly diverse, and obvious phylogenetic signatures have not been observed for archaea that contain mostly leaderless transcripts. For example, the fraction of leaderless transcripts in two species of methanogenic archaea is very low at approximately 11 % in *M. mazei* [9] and 16 % in *Methanolobus psychrophilus* [39]. Similarly, a 12 % fraction of leaderless transcripts has been reported for *Thermococcus kodakarensis* [15], whereas leaderless transcripts have been shown to be nearly absent in *Pyrococcus abyssi* [14]. However, the fraction of leaderless transcripts in the crenarchaeote *Sulfolobus solfataricus* has been shown to be 69 % [10], which is similar to that in the euryarchaeote *Hfx. volcanii*. RNA-Seq studies have not been published for *Pyrobaculum aerophilum*, although in a small-scale study, all of the transcripts were found to be leaderless [40], indicating that this archaeon also contains a high fraction of leaderless transcripts.

The fraction of leaderless transcripts also vary widely in bacteria. For example, leaderless transcripts are very scarce in *Escherichia coli* and might even be confined to specific conditions when a toxin/antitoxin system is induced [41]. In contrast, nearly one-quarter of all *Mycobacterium smegmatis* transcripts are leaderless [42], and 60 % of all transcripts in the radio-tolerant bacterium *Deinococcus deserti* are leaderless [43]. The eukaryotic species *Giardia lamblia* only contains only leaderless transcripts [44].

The occurrence of species with high fractions of leaderless transcripts in all three domains of life and the dependence of leaderless translation on a lower number of initiation factors led to the hypothesis that this mechanism of translation initiation might be the most ancient [45]. This hypothesis is supported by the fact that leaderless transcripts can be translated by ribosomes lacking several ribosomal proteins and the 3′-end of 16S rRNA [46].

#### Leadered transcripts

Next, the length distribution of the 5′-UTRs of the leadered transcripts of *Hfx. volcanii* was analyzed, and the results indicated a wide distribution from 6 to 250 nt (Fig. 2b). Surprisingly, the highest number of these transcripts had lengths from 6 to 20 nt (approximately 60 cases), although this value was much lower than the number from 0 to 5 nt in length (1329 cases) discussed above. The presence of 5′-UTR lengths from 6 to 20 nt was unexpected because shortening the length of a 5′-UTR from 20 to 15 nt for one specific transcript significantly decreased the translational efficiency [38].

The number of transcripts with 5′-UTRs of 21 to 250 nt steadily decreased as the 5′-UTR length increased. Apparently, an optimal 5′-UTR length does not exist in *Hfx. volcanii*, and our results demonstrated that the previously reported 20 nt average length of 5′-UTRs in *Hfx. volcanii* [32] was an underestimation. This conclusion is consistent with the the fact that the number of long 5′-UTRs identified in our study was higher than previously anticipated, although very long UTRs (>150 nucleotides) were infrequent in the present study (only 97 cases).

The lengths of 5′-UTRs differ substantially in other archaea. In *M. mazei*, 5′-UTRs are typically very long, and the majority of 5′-UTRs are 150 to 200 nt [9]. However, most of the 5′-UTRs from *T. kodakarensis* are between 10 and 50 nt and present a median length of 16 nt [15]. The median length of the 5′-UTRs from *P. abyssi* is 37 nt, which is between that of the species mentioned above [14]. The fraction of leadered transcripts in *S. solfataricus* is so low that 5′-UTR lengths have not been reported [10]. Taken together, the high variability in 5′-UTR length indicates that the mechanisms of translation initiation and the importance of translational regulation via 5′-UTRs (e.g., through riboswitches, RNA thermometers, and regulatory sRNAs) may differ widely among archaea. The scarcity of (long) 5′-UTRs in *Hfx. volcanii* identified in the present study indicated that translational control requiring structured leaders is not typical in haloarchaea and that alternative mechanisms potentially based on 3′-UTRs might prevail.

#### Differences between leaderless and leadered transcripts

**Fig. 3:**
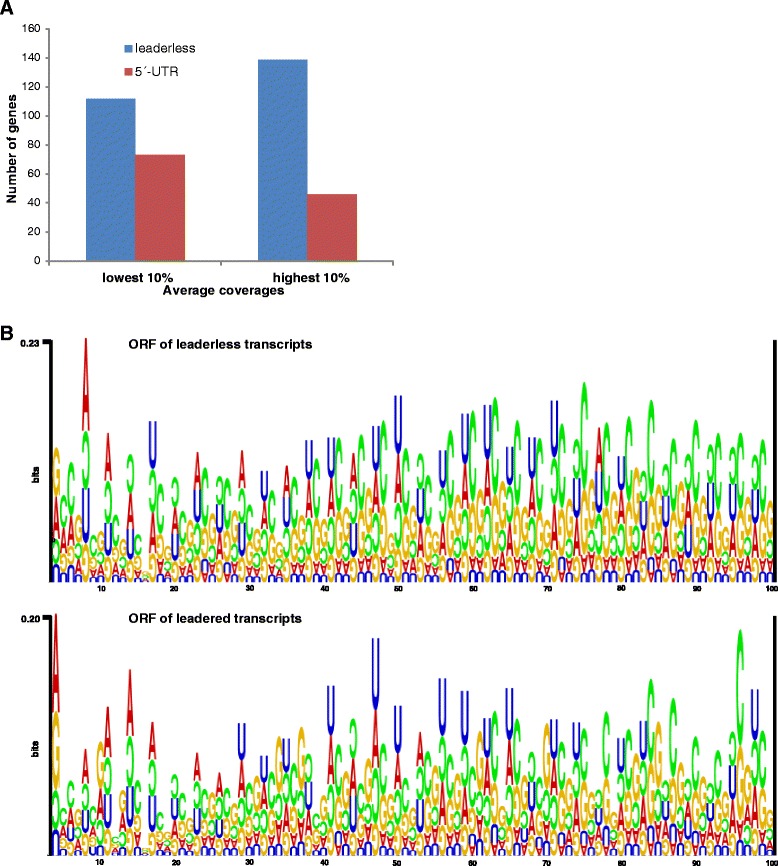
Comparison of leaderless and leadered transcripts. **a** Expression levels of leaderless and leadered transcripts. Number of leaderless (*blue*) and leadered (*red*) genes in the 10 % of transcripts with the lowest average coverage (*left*) and with the highest average coverage (*right*) are shown. **b** Sequence motifs in the ORFs of leaderless (*top*) and leadered (*bottom*) genes. Sequence motifs in the first 97 nt after the start codon in leaderless (*top*) and leadered (*bottom*) transcripts were calculated using “RNA Structure Logo” [47]. The fractions of A, C, G, and U in the two sequence groups were considered. *Upright letters* indicate nucleotide frequencies above the statistical expectation, *bottom up letters* indicate nucleotide frequencies below the statistical expectation. The size of the letters represents the deviation from the statistical expectation

Two approaches were used to address the question of whether systematic differences occur in the expression or sequence bias between leaderless and leadered transcripts. First, the number of leaderless and leadered transcripts for 10 % of the genes with the highest average coverage and for 10 % of the genes with the lowest average coverage was calculated (Fig. 3a). The fraction of leaderless transcripts was much higher for the genes with a high read coverage than for those with a low read coverage, which indicated that highly expressed genes typically have leaderless transcripts. In a second approach for identifying systematic differences between leaderless and leadered transcripts from *Hfx. volcanii*, the sequence logos [47] of the first 100 nt following the start codon were generated for all of the ORFs of the two respective classes (Fig. 3b). Both groups had a much higher A/U content at the second codon position than expected from the overall genome GC content of 67 %. The higher than average A/U content at the second codon position could be explained by the amino acid composition of the *Hfx. volcanii* proteome. In total, *Hfx. volcanii* encoded 4077 proteins containing 1,150,999 amino acids (data not shown). Fifty-three percent of these amino acids were encoded by codons with A or U at the second position, and this rate was considerably higher than the average A/U content of 34 %. However, only 30 % of the amino acids were encoded by codons with A or U at the first codon position, which explained the differences observed at the first versus second codon positions in the sequence logos.

A considerable difference between the leaderless and the leadered transcripts was found for the second codon (the first three positions in Fig. 3b). The prevalence of A/G at the first codon position was much higher in transcripts with a 5′-UTR than in leaderless transcripts. Thus, the amino acid composition at the second position was more restricted in proteins encoded by leadered transcripts. Because the amino-terminal methionine is frequently removed after translation, this penultimate amino acid becomes the amino-terminal amino acid, and its identity could influence the protein half-life. A very slight difference between the leaderless and leadered transcripts was found for the third codon position. Starting at codon six (nt 18), the leaderless transcripts demonstrated a preference for C (and G to a lesser extent), which was somewhat less prevalent for the leadered transcripts (compare Fig. 3b). This result was consistent with the hypothesis that leaderless transcripts in *Hfx. volcanii* are enriched in transcripts with high expression levels and exhibit a higher codon preference compared with leadered transcripts.

#### Start codons of leaderless transcripts

**Fig. 4:**
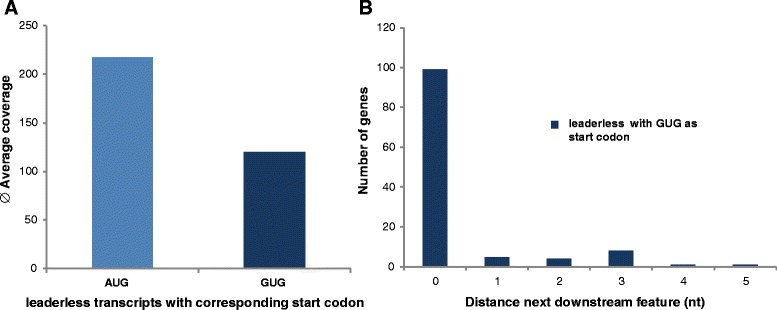
Leaderless transcripts with AUG and GUG start codons. **a** Average coverage of leaderless transcripts with AUG and GUG start codons. The average coverage is shown for all of the leaderless transcripts with a AUG and with a GUG start codon as indicated. **b** Leaderless transcripts with GUG start codon. Number of genes with distances between the experimentally determined TSS and the annotated GTG start codon between zero and five nucleotides are shown

An analysis of the start codons of leaderless transcripts showed that certain transcripts did not have an AUG start codon. We identified 118 leaderless transcripts with GUG start codons, which corresponded to 6 % of all leaderless transcripts. However, the average read coverage of leaderless transcripts with GUG start codons was approximately twofold lower than the average level of leaderless transcripts (Fig. 4a), which indicated that GUG start codons were confined to weakly expressed genes. We also determined that leaderless transcripts with GUG start codons did not require a greater number of additional upstream nucleotides for translation initiation compared with leaderless transcripts with an AUG start codon (Fig. 4b).

The results from an earlier study indicated that leaderless transcripts exclusively require the start codon AUG, whereas leadered transcripts could also use the start codons GUG and UUG [38]. However, this result was based on mutating the AUG codon of only one leaderless transcript to GUG and UUG, which abolished translation initiation. Another study used a translational fusion between the *hsp70* transcript, including a short 4-nt leader with a reporter gene, to analyze the importance of the sequence surrounding the start codon for translation efficiency [48]. Mutating the native AUG codon to a GUG codon led to a decrease in translation efficiency of 83 %. This effect was even more pronounced after removing the short 4-nt 5′-UTR. The translation efficiency of the leaderless version with the GUG start codon was only 2.6 % compared with that of the leaderless version with the AUG start codon, and the authors stated that this low activity could only be quantified because of the high transcript level. Therefore, these studies showed that the translational efficiencies of leaderless transcripts with a GUG start codon were low (2.6 %) or below the detection limit. In each case only one single transcript was used, and neither of these transcripts had native GUG start codons but rather mutated versions of the native AUG start codon.

Importantly, the results of this dRNA-Seq study revealed that a considerable fraction of leaderless transcripts had native GUG start codons (9 % or 118 transcripts); therefore, the results of the two studies described above cannot be generalized to all leaderless transcripts.

#### Protein-coding genes with alternative promoters

For *E. coli*, 10 to 20 % of all genes have been identified as having two transcripts with different 5′-ends, which suggests that the trancripts are transcribed from two different promoters [49]. To analyze whether this pattern might also be true for *Hfx. volcanii*, the possible association of more than one TSS with one gene was examined for the 1851 expressed protein-coding genes. Indeed, 139 protein-coding genes (7.5 %) were associated with two TSSs. Of the 139 genes that were associated with two or three different TSSs, 111 genes had leaderless and leadered transcripts (ten cases had 2 leadered transcripts) and 28 genes had two transcripts (one case had three transcripts) with different 5′-UTR lengths. Interestingly, several transcripts had a leaderless version and an extremely long 5′-UTR version, e.g., HVO_0482 encoding an Hsp20-type chaperone had a leaderless transcript and a transcript with a 5′-UTR of 250 nt. The TSSs of the leaderless transcripts had a high average coverage of 469, whereas the average coverage of the leadered transcripts was considerably lower at 144. Two promoters upstream of one gene may typically have different strengths and different regulatory patterns and thus enhance the regulatory potential of gene expression. One haloarchaeal gene has been long known to be preceded by several promoters, although it is not a protein-coding gene. Namely, the rRNA operon of *H. cutirubrum* has been shown to be preceded by as many as five promoters, all of which are active under optimal conditions but at different strengths (two are hardly detectable). At least one of these promoters has been shown to exhibit differential growth rate-dependent regulation [50]. For *Hfx. volcanii*, the dRNA-Seq results showed three TSSs that were 185, 293, and 444 nt upstream of the 5′-ends of both of the 16S rRNA genes (HVO_1728 and HVO_2939). The first and third TSSs had an extremely high average coverage; therefore, their corresponding promoters might be strong. The distances between TSSs and 16S rRNA genes are different in the two species. The strongest promoter, P1, in *H. cutirubrum* is approximately 750 nt upstream of the 16S rRNA gene. However, this location in *Hfx. Volcanii* cannot be a promoter because 677 nt upstream of the 16 S rRNA gene is the start site of a protein-coding gene on the other strand.

As discussed below, an alternative explanation for the association of two TSSs with one protein-coding gene might be that the upstream TSS belonged to an unannotated small gene that was expressed independently of the downstream gene. However, because of our extensive search for missing gene calls, this occurrence should have been rare.

### Small proteins and uORFs in long 5′-UTRs

In many genomes, the genes encoding small proteins are not annotated to avoid the annotation of many false positives, i.e., sequences that present a certain distance between the start and stop codons but are not real genes because they are neither transcribed nor translated. Often, the lower limit for gene prediction is 100 codons, which results in a systematic disregard for all proteins of less than 11 kDa [51]. However, small proteins do occur in prokaryotes as well as in eukaryotes, and their numbers as well as their importance are heavily underestimated (for reviews, see [52, 53]). For *Halobacterium salinarum*, experimental approaches to isolate and identify small proteins have been optimized, and the “low-molecular-weight proteome” has been characterized [54]. More than 300 small proteins of less than 10 kDa have been identified, and they account for more than 10 % of all proteins, which is a non-negligible fraction.

In the current study, an extensive search for unannotated genes in the *Hfx. volcanii* genome was performed (see Methods) using an approach for detecting conserved (very) short proteins. Thus, complete coverage of conserved genes for small proteins should have been achieved. The TSSs for 291 small protein genes (100 amino acids or less) were detected, which represented 16 % of the transcripts of all protein-coding genes. This result indicated that small proteins form a considerable fraction of the proteome of *Hfx. volcanii* and that a characterization of the biological roles of this neglected class of proteins is urgently required.

In addition to stand-alone genes for small proteins, long 5′-UTRs can contain folded structures, such as riboswitches, and harbor “upstream ORFs” for small proteins (uORFs) [55, 56]. Therefore, the coding potential of all 97 transcripts with very long 5′-UTRs from 150 to 250 nt was analyzed. In 16 cases, ORFs encoding small proteins from 15 to 31 amino acids could be identified (an example is shown in Fig. 1e). All of these proteins had low isoelectric points (typical for haloarchaeal proteins), which suggested that the ORFs were valid genes capable of producing proteins (Table 2). In 7 of the 16 cases, an additional TSS was found upstream of the gene for the normal-sized protein in addition to the TSS upstream of the small protein gene. Therefore, in these cases, the normal-sized genes could be expressed with a very long 5′-UTR containing an uORF and could also be expressed as a leaderless transcript. Alternatively, these cases might be examples of genes encoding small proteins that were situated directly upstream of genes for normal-sized proteins.

**Table 2:** Small proteins encoded in long 5′-UTRs^a^

No	AS	IP	MW	HVO	TSS HVO	gene / FC
1	15	6.7	1705	2055	+	*agl15*
						(prob. low-salt glycan biosyn. flippase)
2	16	5.6	1714	A0265		ISH
3	17	5.8	1896	1842		HY
4	15	6.5	1922	B0328	+	*dppA15* (ABC transport protein)
5	17	6.5	2008	A0121		HTH-10 family transcription regulator
6	17	6.5	2008	A0413		HTH-10 family transcription regulator
7	21	4.2	2205	2018	+	CHY
8	23	5.6	2365	2990	+	HY
9	19	5.9	2386	A0265		ISH
10	23	4.4	2529	A0035A		CHY
11	23	5.6	2640	A0165		SNF family transport protein
12	23	5.4	2743	A0127		tryptophan--tRNA ligase
13	26	5.9	2772	2948		*pheS*
						(phenylalanine-tRNA ligase alpha subunit)
14	24	5.9	2966	2432	+	ABC transport protein
15	27	5.9	3206	A0442	+	CcbP family protein
16	31	5.4	3363	B0198	+	ABC transport protein

^a^Protein lengths (No. amino acids, AS), isoelectric points (IP) molecular masses (MW), gene designations (HVO_NNNN), occurrences of an additional TSS at the normal-sized ORF, and function of the protein of the normal-sized ORF are shown

### Previously known non-coding RNAs

#### Stable RNAs

In addition to protein-coding genes, the TSSs for previously known RNA genes were also analyzed (Table 1, Additional file 2: Table S2). The TSSs were detected for RNase P RNA and for 7S RNA, which is part of the signal recognition particle. *Hfx. volcanii* encodes a type I-B CRISPR-Cas system, and TSSs were detected for all three CRISPR RNAs. The previously observed deletion of the CRISPR locus P1 could be confirmed because reads were not obtained for this region [57, 58]. We found reads for all of the other spacers, which again confirmed earlier observations [57]. Consistent with the generation of CRISPR RNAs via the processing of large primary transcripts, their reads were not enriched in the +TEX library and did not constitute TSSs.

The TSSs for both ribosomal operons were found upstream of the 16S rRNA. However, as expected, no TSSs were identified for the mature 5S rRNAs, 16S rRNAs, and 23S rRNAs from the two rRNA operons because these rRNAs were processed from a long primary rRNA operon transcript. The two genes for tRNA^Ala^ that were associated with the two rRNA operons and the gene for tRNA^Cys^ that was encoded in one of the two ribosomal operons were also not associated with a TSS.

All of the tRNAs reads were detected in the –TEX libraries, showing that all of the tRNAs were transcribed, although the TSSs in close proximity to the mature tRNA 5′-end were only found for 39 of the 52 tRNA genes. The lack of a proximal TSS for the remaining 13 tRNA genes could be explained by different mechanisms: 1) in three cases, the tRNA gene was part of an rRNA operon (see above); 2) in four cases, two tRNA genes formed bicistronic operons (HVO_0345- > HVO_0344, HVO0523- > HVO_0524, HVO_2457- > HVO_2458, and HVO_2566- > HVO_2567), and the first but not the second gene was associated with a TSS; 3) one primary tRNA transcript had an exceptionally long leader of 300 nt (tRNA^Gly^CCC); 4) the tRNA might be part of a multicistronic operon, which could be true for tRNA^Gly^TCC; and 5) because tRNAs fold into stable structures, the preparation of cDNAs from these molecules was not trivial; therefore, certain TSSs may have been missing because of technical problems that occurred during library preparation.

#### Previously known sRNAs

In *Hfx. volcanii*, an earlier RNA-Seq approach identified 145 intergenic sRNAs and 45 cis-antisense sRNAs [11]. Sixty-two of these sRNAs were also associated with TSSs in this dRNA-Seq analysis. The lack of identification of all previously known sRNAs may be explained as follows. First, in the earlier study, the cells were not only grown under optimal conditions, but cultures from six conditions were analyzed; many of the sRNAs were detected solely in cells grown under non-optimal conditions, such as low salt conditions [11]. Second, the sRNAs that were not primary transcripts but that were generated by processing were not included in the TSS analysis of this dRNA-Seq study.

### Novel transcripts

**Fig. 5:**
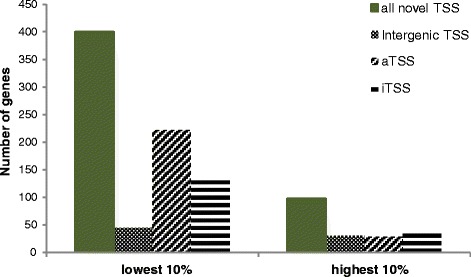
Number of novel genes with very low or very high transcript levels. The number of all novel transcripts and the three subclasses, intergenic TSS, iTSS, and aTSS, present in the 10 % of the transcripts with the lowest and highest average coverages are shown

In total, 2792 novel TSSs were detected, which corresponded to 59 % of all TSSs (Table 1). The number of novel transcripts was much higher in the 10 % of transcripts with the lowest coverage than in the 10 % with the highest coverage, which indicated that the intracellular levels of the majority of the novel transcripts were low (Fig. 5). The present study could not provide information about the size of the transcripts because of the restriction to the 5′-ends of the transcripts. Therefore, it is unclear how many of the novel transcripts were valid sRNAs and how many represented other types of RNAs, e.g., protein-coding RNAs. However, the six-frame translation of the *Hfx. volcanii* genome was compared with the UniProt database (see Methods); therefore, the number of missing gene calls should be very low. The novel transcripts were subdivided into intergenic transcripts (TSS), antisense transcripts (aTSS), and internal transcripts (iTSS). Examples of these classes are shown in Fig. 1c and d. The three classes of novel transcripts are discussed in detail below.

#### Novel intergenic RNAs

In this study, 395 novel intergenic RNAs that had a minimal distance of at least 250 nt to the start of any known gene were identified. Because UTRs become less frequent with increasing length and the longest previously determined haloarchaeal 5′-UTR had a length of 145 nt [32], it is unlikely that many of these TSSs were associated with protein-coding genes with extremely long leaders. Because of the limited size of the intergenic regions, most or all of these novel transcripts were most likely small and could be added to the class of intergenic sRNAs, thereby increasing the number of intergenic sRNAs from 145 to 540.

#### Novel internal RNAs (iTSS)

In total, 1153 novel internal TSSs were detected. In a former study, internal transcripts were not discussed because they might represent meta-stable degradation intermediates without biological functions [11]. However, enrichment in the TEX-treated sample indicated that all of the iTSSs that were detected in this study were valid primary transcripts.

The iTSS class likely contained spurious transcripts that were initiated by the RNA polymerase “by mistake” and that lacked any biological function. An increase in the sequencing depth leads to an increase in the identification of real transcripts but also leads to an increase in the identification of spurious transcripts [59]. However, a considerable fraction of iTSSs were most likely transcripts of biological relevance because 51 % were preceded by a basal promoter and only 49 % were not preceded by a recognizable promoter (promoter motifs are discussed below). Thus, the identified iTSSs probably included a large fraction of transcripts of biological relevance. However, experimental validation is required to determine the importance of individual iTSSs.

iTSSs that are localized close to the 3′-ends of proximal genes on polycistronic transcripts might represent additional transcription initiation sites for distal genes driven by promoters localized within the proximal genes. In the current study, an analysis of the iTSSs located at the 3′-end of annotated genes showed that 144 (12 %) of the 1153 iTSSs had annotated genes downstream (distance 1 to 250 nt); thus, these iTSSs most likely represented promoters for a downstream gene. One example of an internal promoter for a downstream gene was recently reported for *Hfx. volcanii* [60]. The overlapping gene pair HVO_2723/HVO_2722 was transcribed from a promoter upstream of the first gene, leading to a bi-cistronic mRNA. In addition, the second gene was expressed separately from a promoter within the first gene. A similar example is shown in Fig. 1f. Additionally, for *Halobacterium salinarum*, a large number of internal transcripts have been described, and they are preceded by transcription factor-binding motifs; thus, they have been proposed as primary transcripts [61].

A special class of iTSSs accounted for the internal transcripts that overlapped with the 3′-end of transposase genes. The overexpression of one of these internal transcripts in *H. salinarum* has been shown to result in improved growth, indicating that these 3′-end overlapping internal RNAs have regulatory functions [62]. In the present study, five novel internal RNAs overlapped with the 3′-ends of transposase genes, which indicated that a regulatory function for such RNAs is not confined to *Halobacterium*. For one of these cases, HVO_0008, the internal overlapping RNA was verified by Northern blot analysis (data not shown). A further 41 novel internal transcripts overlapped with transposase genes, albeit not with the 3′-end. The novel internal transcripts that overlapped with transposase genes might be important for controlling the transposition frequency, which was supported by the extremely low average coverage of the respective transposase transcripts in all but four cases and the lack of TSS detection for these genes in this dRNA-Seq study. Internal transcripts overlapping transposase genes have also been found in *S. solfataricus* [63]*, T. kodakarensis* [15] and *Pyrococcus abyssi* [64], which indicates a widespread regulatory mechanism in archaea.

#### Novel antisense RNAs (aTSS)

**Fig. 6:**
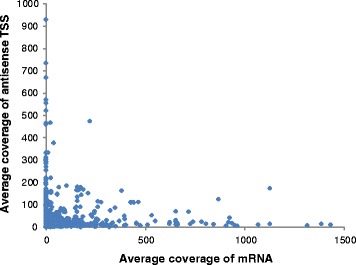
Reverse correlation of transcript levels of antisense transcript and their cognate sense transcripts. A scatterplot showing the average coverages of antisense transcripts and their cognate sense transcripts

The largest class of novel transcripts was formed by 1244 antisense transcripts. Most of these transcripts were antisense to protein-coding genes, although six were antisense to previously known sRNAs. For 60 % of the antisense transcripts, the corresponding sense transcript could not be detected by this dRNA-Seq approach, indicating that the antisense transcripts might down-regulate the expression of the transcript on the opposite strand. This hypothesis was supported by plotting the average coverages of antisense transcripts against the average coverages of the cognate sense transcripts (Fig. 6). With few exceptions, nearly all of the data points were near or on top of one of the two axes, which indicated that the concentrations of sense and antisense transcripts were reciprocal and that antisense transcripts might down-regulate the concentrations of their cognate sense transcripts. However, further experimental evidence is required to clarify the regulatory role of antisense RNAs in *Hfx. volcanii*. The lengths of antisense RNAs must also be determined because the dRNA-Seq results did not contain any length information because of read length restrictions of approximately 100 nt. The analysis of all 1244 aTSSs revealed that 50 (4 %) had annotated genes downstream from 1 to 250 bp and might represent promoters for a gene downstream of the aTSS and closely upstream of the sense gene.

The presence of a high fraction of antisense transcripts is not confined to *Hfx. volcanii* but appears to be widespread among many phylogenetic groups. For example, high fractions of antisense RNAs have been described for higher eukaryotes (reviews: [65–69]). In bacteria, the fractions of antisense transcripts vary widely from 1 to 2 % in *Salmonella* to more than 50 % in *Staphylococcus aureus* to approximately 75 % in *Prochlorococcus* strains (reviews: [6, 70–73]). High levels of antisense transcripts have also been observed in other archaea, e.g., 26 % in *Methanolobus psychrophilus* [16] and *Pyrococcus abyssi* [14].

In bacteria, antisense transcripts are involved in the regulation of transposition [73]. This involvement appeared to hold true for *Hfx. volcanii* because 134 of the 1244 antisense transcripts overlapped with transposase genes. The presence of antisense transcripts to transposons has also been reported for other archaea, e.g., *T. kodakarensis* [15].

The results of the dRNA-Seq approach employed here showed that during exponential growth under optimal conditions, antisense transcripts were present in approximately 30 % of all of the protein-coding genes.

#### Distribution of iTSSs and aTSSs within ORFs

**Fig. 7:**
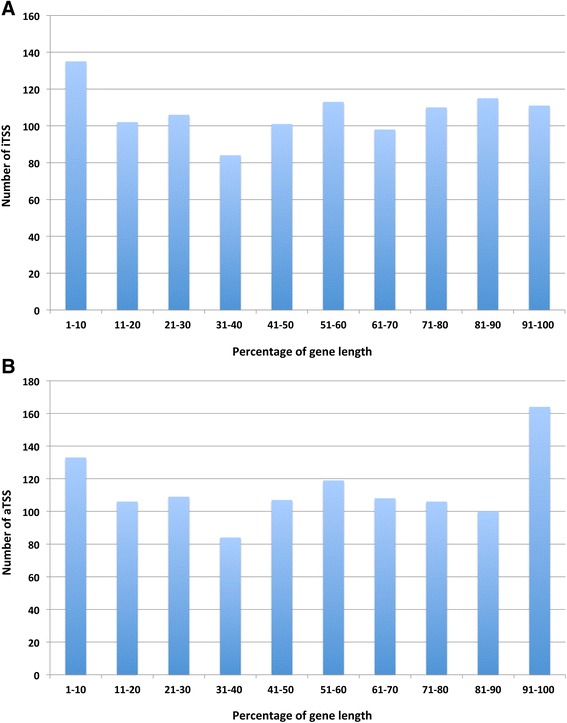
Intragenic distribution of iTSS and aTSS. **a** Location of iTSSs in annotated genes. Genes for which an iTSS in sense was detected were divided into 10 equal-sized sections (x-axis). The number of iTSS in each section was determined (y-axis). **b** Location of aTSSs in annotated genes. Genes for which an aTSS was detected were divided into 10 equal-sized sections (x-axis). The number of aTSSs in each section was determined (y-axis)

To further analyze the locations of the TSSs internal or antisense to annotated genes, the genes were divided into 10 equal sections and the numbers of iTSSs and aTSSs per section were separately determined (Fig. 7). According to this analysis, the iTSSs were equally distributed across genes with a slight preference for the 5′-end, whereas the aTSSs had a slight preference for the 3′-end. A similar distribution for iTSSs has been found in *E. coli* [74] and *M. psychrophilus* [16], whereas both the iTSSs and aTSSs have been shown to be preferably located at both the 5′- and 3′-ends in *Shewanella oneidensis* [75].

### Promoter structure

Transcription initiation in archaea and transcription initiation in eukaryotes share homologous basal transcription factors and transcription factor-binding motifs. Species of both domains contain the “TATA box binding protein” (TBP) and TATA boxes as well as “transcription factor (II) B” (TFB) and the transcription factor B recognition element (BRE) [76–78]. However, the consensus sequences for the basal promoter elements are not identical between archaea and eukaryotes, and even within the archaea, different groups exhibit different consensus sequences [79]. For haloarchaea, the motifs “TTWT” (W = A or T) for the TATA box, “CGAAA” for the BRE, and “WW” for a −10 element have been proposed [32]. We used the information obtained from the upstream regions of the 4749 experimentally determined TSSs to increase the quality of promoter predictions and develop a “promoter score” as a quantitative value to determine how close each individual upstream region fits with a genome-wide consensus. To this end, the regions upstream of all of the TSSs were analyzed to generate a position weight matrix (PWM) that quantified the non-randomness of a subregion of 28 nt around the known promoter elements BRE and the TATA box. The initial PWM was improved using an iterative approach that utilizes a PWM-Scan routine implemented in the MOODS software package (for details, see the Methods) [80]. The final PWM was used to calculate a promoter score for every TSS and its position relative to the TSS, and this information is included in Additional file 2: Table S2.

**Fig. 8:**
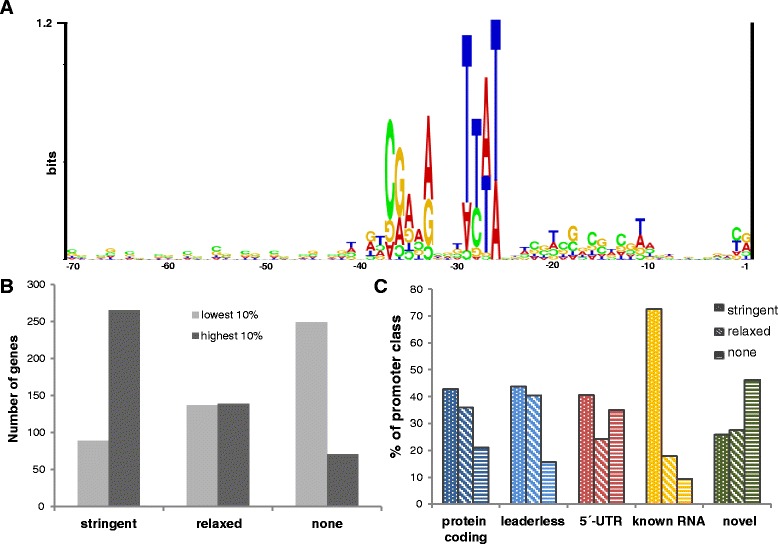
Promoter analysis. **a** Motifs of basal promoter elements. A position weight matrix (PWM) was generated and used to calculate a promoter score for each gene. An RNA structure logo was generated from the 28-mers that resulted in the final position weight matrix (PWM). This logo was generated from 1302 sequences with a MOODS search score below the threshold of 0.001. **b** Expression of genes preceded by a stringent, a relaxed, or no recognizable promoter. The numbers of genes preceded by a stringent, a relaxed, or no recognizable promotor that were present in the groups of transcripts with the 10 % highest (*dark grey*) and the 10 % lowest (*light grey*) average coverage. **c** Fractions of stringent and relaxed promoters preceding different classes of genes. Genes with a promoter score above 4.7 (*p* ≤ 0.001) had a stringent promoter, genes with a promoter score between 1.2 and 4.6 (*p* ≤ 0.01) had a relaxed promoter. The fractions of genes preceded by a stringent, relaxed, or no recognizable promoter were calculated for the indicated classes of genes

To evaluate the average predictive power of the promoter score, three classes were defined as follows: genes were classified as having a “stringent promoter” when the MOODS search had a match exceeding the significance threshold of *p* = 0.001 (which was true for promoter scores higher than 4.7), having a “relaxed promoter” when a match exceeded the significance threshold of *p* = 0.01 (scores between 1.2 and 4.6), and having “no promoter” when no match was reported by the MOODS search. A structure logo [47] of the promoter region is shown in Fig. 8a, which clearly shows the basal promoter elements BRE and the TATA box.

Using these definitions, 34 % of all TSSs were preceded by a stringent promoter and 31 % were preceded by a relaxed promoter; however, 55 % of all TSSs were not preceded by any basal promoter elements. Several lines of evidence revealed that the average predictive value of the promoter score was high. For example, the average coverage of genes with stringent promoters was considerably higher than that for genes with relaxed promoters or no promoters (296 versus 137 versus 71, respectively). In addition, the types of promoters upstream of the 10 % of transcripts with the highest and lowest numbers of reads were analyzed. The majority of genes with high transcript levels were preceded by a stringent promoter, whereas most genes with low transcript levels were not preceded by stringent or relaxed promoters (Fig. 8b). Next, the promoter distributions upstream of the different classes of transcripts were analyzed (Fig. 8c). Nearly all of the previously known genes for non-coding RNAs were preceded by a stringent promoter, indicating that their transcription was initiated using the same basal transcription factors as those used for protein-coding genes. Approximately 80 % of all protein-coding genes were preceded by a stringent or relaxed promoter, and this percentage was somewhat higher for leaderless than for leadered transcripts. However, only a small fraction of the novel transcripts were preceded by a stringent promoter, which is consistent with the high sequencing depth of this dRNA-Seq approach that was required for their discovery.

## Conclusions

This dRNA-Seq analysis of the *Hfx. volcanii* transcriptome led to the detection of 4749 TSS candidates, 2792 of which had not been previously observed and thus represented novel transcripts. Only 1851 of the 4749 TSSs belonged to protein-coding genes; therefore more than 60 % of all TSS belonged to transcripts that were likely non-coding. The latter fraction was unexpectedly high, which presented a new understanding of the *Hfx. volcanii* transcriptome. Seventy-two percent of the protein-coding transcripts were leaderless, which underscored the fact that the leaderless mRNA pathway was the principal method by which translation was initiated in *Hfx. volcanii*. The 5′-UTRs of the remaining 28 % of protein-coding transcripts had a very broad length distribution, and we could not identify an optimal 5′-UTR length. A significant number of antisense RNAs was identified (1244 aTSSs), and these constituted the highest fraction of non-coding RNAs. A strong negative correlation between the coverage of antisense RNAs and the cognate sense mRNAs was observed, which suggested that the downregulation of gene expression via antisense RNAs may be more important than previously anticipated. In addition, a high number of iTSSs was detected, and a fraction of these iTSSs might represent sub-operonic transcripts of downstream genes in polycistronic operons. The identified iTSSs and aTSSs add to and highly outnumber the as yet best studied class of non-coding RNAs: intergenic sRNAs. In summary, this dRNA-Seq analysis yielded a comprehensive overview of the coding and non-coding fractions of the *Hfx. volcanii* primary transcriptome.

## Methods

### Archaeal strains

The *Hfx. volcanii* strains H26 and H119 [31] were chosen for this study because they are widely used by many laboratories and because targeted mutations can easily be introduced into their genomes. H26 was derived in two steps from the *Hfx. volcanii* wildtype DS2. H26 was cured from the small plasmid pHV2 and has a deletion in the *pyrE* gene, which encodes an enzyme essential for uracil biosynthesis. H119 was derived from H26 and has deletions in the two amino acid biosynthesis genes *trpA* and *leuB*. None of these mutations were relevant to this dRNA-Seq study because the cultures were grown in complex medium.

### Construction of cDNA libraries and Illumina sequencing

Three independent cultures were performed with slight differences in the library preparation. In each case, part of the sample was treated with TEX (+TEX) and part of the sample remained untreated (−TEX). Therefore, six samples were analyzed by high-throughput sequencing.

#### Replicate 1: Frankfurt −/+TEX

H26 was grown in complex medium under optimal conditions as previously described [81]. Briefly, the optimal conditions included a temperature of 42 °C, a NaCl concentration of 2.2 M, and good aeration (shaking at 250 rpm and 30-ml culture volume in 100-ml Erlenmeyer flasks). Cell growth was monitored spectroscopically (OD_600_) and by quantifying the cell densities using a Neubauer counting chamber. The cultures were inoculated with exponentially growing pre-cultures and grown to the mid-exponential growth phase (4–5 × 10^8^ cells/ml) before they were harvested and used for the RNA-Seq analysis. The cultures were harvested by centrifugation, and total RNA was isolated using the RNeasy kit according to the manufacturer’s instructions, which included an extensive DNase treatment (Qiagen, Hilden, Germany). The RNA integrity was assessed by analytical gel electrophoresis, the absence of DNA was verified by comparing PCR reactions with and without a prior cDNA step, and the nucleic acid concentration was determined using a NanoDrop 1000 photometer (Thermo Fisher Scientific). Enzymatic treatment with TEX and tobacco acid pyrophosphatase (TAP), RNA adaptor ligation with T4-RNA-ligase, and cDNA synthesis were performed as previously described [82]. Illumina sequencing was performed on a HiSeq 2000 with 100 cycles in single-end mode at the Max Planck Genome Center in Cologne, Germany.

#### Replicate 2: Würzburg −/+TEX

H26 cultures were grown, and total RNA was isolated in Frankfurt as described above. Libraries for Illumina cDNA sequencing were prepared by Vertis Biotechnology AG, Germany (http://www.vertis-biotech.com/) as previously described for eukaryotic microRNAs [83] but without the RNA size-fractionation step prior to the cDNA synthesis. The transcripts were not fragmented to obtain sequencing reads from the 5′-transcript ends. Equal amounts of RNA samples were poly(A)-tailed using poly(A) polymerase. Then, the 5′-triphosphates were removed by applying tobacco acid pyrophosphatase (TAP), which resulted in 5′-monophosphates. Afterwards, an RNA adapter was ligated to the 5′-phosphate of the RNA. First-strand cDNA was synthesized using an oligo(dT)-adapter primer and M-MLV reverse transcriptase. In a PCR-based amplification step using a high-fidelity DNA polymerase, the cDNA concentration increased to 20 to 30 ng/μl. A library-specific barcode for multiplex sequencing was part of a 3′-sequencing adapter. The following adapter sequences flanked the cDNA inserts:

TrueSeq Sense primer

5′AATGATACGGCGACCACCGAGATCTACACTCTTTCCCTACACGACGCTCTTCCGATCT-3′

TrueSeq Antisense NNNNNN primer (NNNNNN = 6n barcode for multiplexing)

5′-CAAGCAGAAGACGGCATACGAGAT-NNNNNN-GTGACTGGAGTTCAGACGTGTGCTCTTCCGATC(dT25)-3′

The resulting cDNA libraries were sequenced using a HiSeq 2000 machine (Illumina) in single-read mode and running 100 cycles.

#### Replicate 3: Ulm −/+TEX

*Haloferax* cultures (H119) were grown in the Marchfelder Laboratory in Ulm in Hv-YPC medium [31] to an OD_600_ of 0.8. RNA was isolated and divided into two RNA fractions: fractions 1 and 2. From fraction 2, the rRNA was removed using the RiboZero kit (Epicentre). Both RNA fractions were subsequently sent to Vertis (Vertis Biotechnologie AG, Martinsried, Germany) for cDNA preparation and high-throughput sequencing. Here, RNA fraction 1 was incubated with TEX to remove the RNA species that carried a 5′-mono-phosphate. Thus, this RNA fraction was enriched for primary transcripts (fraction +TEX). For cDNA synthesis, both RNA fractions were poly(A)-tailed using poly(A) polymerase and then treated with TAP to remove the 5′-PPP structures. Afterwards, an RNA adapter was ligated to the 5′-monophosphates of the RNAs. First-strand cDNA synthesis was performed using an oligo(dT)-adapter primer and M-MLV reverse transcriptase. The resulting cDNAs were PCR-amplified to approximately 10 to 20 ng/μl using a high-fidelity DNA polymerase. Barcode sequences, which were part of the 3′-sequencing adapter, were added as the PCR cycles completed. The cDNAs were purified using the Agencourt AMPure XP kit (Beckman Coulter Genomics) and then analyzed by capillary electrophoresis. For Illumina sequencing, the cDNAs were pooled in approximately equimolar amounts. The cDNA pool was eluted in the size range of 200 to 500 bp from a preparative agarose gel. An aliquot of the size-fractionated cDNA pool was analyzed by capillary electrophoresis. The cDNA pool was sequenced on an Illumina HiSeq 2000 machine with a 100-bp read length.

### Reference genome reannotation

The curated *Hfx. volcanii* genome from the HaloLex database with an up-to-date annotation [84, 85] was used.

At an early stage of the analysis, we systematically analyzed all of the TSSs that were internal to protein-coding genes but close to start codons (within 250 bp). The corresponding proteins were subjected to homology-based start codon checking [85]. In several cases, this manual curation effort allowed us to detect and correct start codon assignment errors. Commonly, the TSS was upstream of the corrected start codon. This analysis was completed prior to the final evaluation of the TSS data.

In the context of the current manuscript, an additional approach was developed to identify missing gene calls. In particular, even very short protein-coding genes could be included in the genome annotation after completing this procedure. This analysis was also completed prior to final TSS mapping. All of the intergenic regions (defined as not-overlapping protein-coding genes, stable RNAs or transposons) were identified, sorted by length, and concatenated using a 12-mer linker (TTAATTAATTAA). This linker introduced stop codons in all six reading frames. Chunks of this concatenated sequence (2–3 kb) were analyzed against UniProtKB (i.e., SwissProt plus TrEMBL) using the blast service at UniProt (which executes BlastX with nucleotide sequences). All of the subregions with strong blast matches were manually evaluated. Several previously missing gene calls were identified by this approach. A large number of strong blast matches were false positives because of invalid start codon assignments (database ORFs were too long) or the presence in the draft genome sequences of pseudogenes, which frequently have aberrant C-terminal sequences. A large number of *Haloferax* genomes have been sequenced [86, 87], and some are extremely similar to *Hfx. volcanii* at the DNA sequence level. By the approach described here, all of the ORF calls produced during those genome sequencing projects were automatically reconciled with the *Hfx. volcanii* reference genome. All of the previously determined sRNAs genes [11, 33, 34] were added to the annotation.

### Read mapping and prediction of transcription start sites (TSSs)

The Illumina reads in FASTQ format were trimmed with a cut-off Phred score of 20 by the program fastq_quality_trimmer from the FASTX toolkit version 0.0.13 (http://hannonlab.cshl.edu/fastx_toolkit/). The following analysis steps were performed using the subcommand “create”, “align” and “coverage” of the tool READemption version 0.3.5 [88]. The poly(A)-tail sequences were removed, and a size-filtering step was applied in which sequences shorter than 12 nt were discarded. The collections of the remaining reads were mapped to the reference genome sequences using segemehl and its remapper lack version 0.2.0 [89]. Mapping statistics (input, aligned, uniquely aligned reads, etc.) can be found in Additional file 1: Table S1. Coverage plots in wiggle format representing the number of aligned reads per nucleotide were generated based on the mapping files and visualized in the Integrated Genome Browser [90]. Each graph was normalized to the total number of reads that could be aligned from the respective library. To restore the original data range, each graph was then multiplied by the minimum number of mapped reads calculated among all libraries.

The TSSs were predicted based on the normalized coverage files obtained from TSSpredator version 1.06 [82] using the “super specific” parameter set but with a minimal enrichment factor of 2.5 instead of 3.0.

The read mapping data underlying the detected TSSs were visually inspected using the read mapping viewer ReadXplorer [91].

The TSSs of all of the replicons were loaded into one Excel table with accompanying information, such as the replicon identity, localization, coverage in the six datasets, associated genes, etc. Very closely spaced TSSs with a distance of less than 10 nt were considered one TSS. The TSSs were sorted into different classes using the following parameter: all of the TSS were annotated as “novel genes” when the next annotated gene start site was more than 250 nt away. If the TSS was found within an annotated gene, the transcripts were classified as antisense transcripts or internal transcripts depending on the strand of TSS and gene. For known RNAs (stable RNAs and sRNAs), a distance between −3 (retraction by 3) and +250 nt was allowed. A small retraction was assigned for a few sRNAs that seemed to be annotated as slightly too long. For protein-coding genes, TSSs with an annotated gene start 0 to 5 nt downstream were classified as “leaderless transcripts” and TSSs with an annotated gene start 6 to 250 nt downstream were classified as “leadered transcripts”.

### Analysis of iTSS and aTSS distributions within open reading frames

Annotated genes for which an iTSS was detected were split into 10 equal-sized sections, where the first section covered the first 10 % of the gene, the second section covered the second 10 %, etc. The number of iTSSs for each section was determined for all genes.

### Promoter analysis

Each TSS was associated with a sequence of 71 bases, and the last base corresponded to the first base of the transcript. We used a fragment of 28 bases centered around the TATA box and preceding the BRE element to calculate a position weight matrix (PWM). Each 71 mer was scored against this PWM using the search function from the MOODS package [79]. The parameters were threshold = 0.001, threshold_from_p = True, convert_log_odds = True, and both_strands = False. The background base composition was calculated from the complete genome and not from the collection of 71-mers. The MOODS search reported hits above the applied threshold, which we considered rather restrictive, and their match position. A prominent match position maximum was also observed (47 % of all positives assigned to that position and 78 % if the adjacent position on each side was also considered). There was a degree of flexibility, albeit restricted, for the distance between the TATA box and the transcription start; therefore, the TATA boxes may have been misaligned when sequence phasing was based on the transcription start site. An iteration was started where only the search hits above the threshold were retained and a slight deviation (4 bases) from the mean position was allowed. Sequences with match positions only few nucleotides from the peak positions were shifted accordingly. A new PWM was calculated from this subset and re-analyzed by a MOODS search against the complete set of 71-mers. As the PWM evolved, previously MOODS-negative upstream sequences became positive and vice versa. After 43 iterations, we obtained complete convergence. The main convergence occurred by the 25th iteration, and thereafter, fewer than 10 sequences changed between subsequent iterations. The resulting 1302 sequences were used to generate an RNA structure logo [47].

The final PWM was used for a MOODS search with threshold of 0.001 against the original non-shifted 71-mers, and the score and match position were recorded. This process resulted in “stringent matches”. Then, the MOODS search was repeated with a relaxed threshold of 0.01 to calculate the “relaxed matches”.

## Abbreviations

dRNA-SeQ, differential RNA-Seq; PWM, position weight matrix; TEX, 5’-P-dependent terminal exonuclease; UTR, untranslated region

## Additional files

Additional file 1: Table S1. Summarizing the statistics of sequencing and read mapping. For each of the six sequencing runs (three with, three without TEX treatment) the number of reads, the number of mapped reads, etc. are tabulated. (XLSX 9 kb) Additional file 2: Table S2. Containing the dRNA-Seq results. Summary of all of the TSSs identified by the dRNA-Seq approach and the additional selected parameters, e.g., replicon, localization, strand, identification in the three datasets, TSS class, associated gene, and promoter score. The first Excel sheet contains the results, and the second Excel sheet contains a list of columns and definitions. (XLSX 572 kb)
